# Comparative Transcriptomics Reveals the Key lncRNA and mRNA of Sunite Sheep Adrenal Gland Affecting Seasonal Reproduction

**DOI:** 10.3389/fvets.2022.816241

**Published:** 2022-04-08

**Authors:** Xiaolong Du, Xiaoyun He, Qiuyue Liu, Ran Di, Qingqing Liu, Mingxing Chu

**Affiliations:** ^1^Key Laboratory of Animal Genetics, Breeding and Reproduction of Ministry of Agriculture and Rural Affairs, Institute of Animal Science, Chinese Academy of Agricultural Sciences, Beijing, China; ^2^College of Animal Science and Technology, Anhui Agricultural University, Hefei, China

**Keywords:** HPA axis, seasonality, photoperiod, candidate gene, sheep

## Abstract

The hypothalamic–pituitary–adrenal (HPA) axis plays an important role in the growth and development of mammals. Recently, lncRNA transcripts have emerged as an area of importance in sheep photoperiod and seasonal estrus studies. This research aims to identify lncRNA and mRNA that are differentially expressed in the sheep adrenal gland in long (LP) or short (SP) photoperiods using transcriptome sequencing and bioinformatics analysis based on the OVX + E_2_ (Bilateral ovariectomy and estradiol-implanted) model. We found significant differences in the expression of lncRNAs in LP42 (where LP is for 42 days) vs. SP-LP42 (where SP is for 42 days followed by LP for 42 days) (*n* = 304), SP42 (where SP is for 42 days) vs. SP-LP42 (*n* = 1,110) and SP42 vs. LP42 (*n* = 928). Cluster analysis and enrichment analysis identified SP42 vs. LP42 as a comparable group of interest and found the following candidate genes related to reproductive phenotype: *FGF16, PLGF, CDKN1A, SEMA7A, EDG1, CACNA1C* and *ADCY5*. *FGF16* (Up-regulated lncRNA MSTRG.242136 and MSTRG.236582) is the only up-regulated gene that is closely related to oocyte maturation. However, *EDG1* (Down-regulated lncRNA MSTRG.43609) and CACNA1C may be related to precocious puberty in sheep. *PLGF* (Down-regulated lncRNA MSTRG.146618 and MSTRG.247208) and *CDKN1A* (Up-regulated lncRNA MSTRG.203610 and MSTRG.129663) are involved in the growth and differentiation of placental and retinal vessels, and *SEMA7A* (Up-regulated lncRNA MSTRG.250579) is essential for the development of gonadotropin-releasing hormone (GnRH) neurons. These results identify novel candidate genes that may regulate sheep seasonality and may lead to new methods for the management of sheep reproduction. This study provides a basis for further explanation of the basic molecular mechanism of the adrenal gland, but also provides a new idea for a comprehensive understanding of seasonal estrus characteristics in Sunite sheep.

## Introduction

Animals that show seasonal reproduction patterns only mate at certain times as their reproductive cycles start and stop based on the season (1, 2). Sheep are seasonal breeders and are often used as a model species to study the effect of photoperiod on reproductive function (2–4). The reproductive endocrine axis of ewes is affected by variations in photoperiod. Ewes transition from an estrus state to an anestrus state from spring to autumn (5, 6). Light affects the secretion of melatonin, which leads to changes in the circadian rhythm of seasonal reproduction of animals (7). Melatonin is produced by the pineal gland, which then acts on the hypothalamus, affecting sheep reproduction through the hypothalamic–pituitary–gonadal (HPG) axis (8–11). The HPG axis and HPA axis are closely related and influence each other. For example, the PVN (paraventricular nucleus) is stimulated to secrete corticotropin-releasing hormone (CRH) which then activates the release of adrenocorticotropic hormone (ACTH) from the pituitary. The ACTH, in turn, stimulates secrete of cortisol from the adrenals which then provide negative feedback back to the brain in a classic homeostatic feedback loop to fine-tune HPA axis signaling (12). Vast quantities of studies show that basal cortisol levels are higher in females than males and the capacity of glucocorticoid secrete was higher in females, suggesting that E_2_ (17β-estradiol) maybe increases HPA axis reactivity (13).

Estrogen is one of the most important hormones in sheep reproduction, especially in anestrus animals. Estrogen negatively regulates the neuroendocrine circuit, affecting the secretion of GnRH (14, 15). Meanwhile, the E_2_-induced surge pattern of luteinizing hormone (LH) and GnRH secretion that conducts ovulation in females, is assailable to the effects of cortisol (12). However, the exact molecular mechanism is not clear. A previous study by Luo et al. (16) found exogenous cortisol treatment of gonad-intact female mice restrained cyclicity in diestrus. Ovariectomy (OVX) female mice were treated with an LH surge-inducing E_2_ implant, as well as a cortisol or cholesterol (control) pellet, and detected two days later for LH levels on the prospective LH surge. All cholesterol-treated females showed a clear LH surge, whereas LH levels were undetectable in cortisol-treated females (16). Many experiments have shown that glucocorticoids can affect the related function of LH. Such as cortisol after infusion of encephalocoele suppresses LH pulse amplitude in ovariectomized ewes (17). At present, most researchers use hypothalamic–pituitary disconnection (HPD) model to study the effect of photoperiod on sheep reproduction, and it has been proved that prolactin is a key hormone involved in the seasonal reproduction of sheep (18–20). It has also been proved that OVX + E_2_ model is also a classical model for the study of photoperiod regulation and hypothalamic function (21, 22).

The rapid development of RNA-seq technology has improved the efficiency of animal molecular genetics and breeding. Long-stranded, non-coding RNA (lncRNA) is a non-coding RNA with a length of more than 200 bp (23, 24). Studies have shown that lncRNA regulates many biological functions, including, dose compensation effect, epigenetics and cell differentiation (24). The topic has become a research hotspot across multiple scientific disciplines, and many lncRNAs have been associated with animal reproduction. For example, several lncRNAs have been associated with STH (Small-tailed Han sheep) fertility (25) and adolescent development in the hypothalamus of goats (26, 27). Moreover, analysis of the hypophysis of Hu sheep with high and low fertility identified 57 differentially expressed lncRNAs (28). These studies show that lncRNAs in the pituitary, and ovaries, of sheep have regulatory functions in reproduction (29). The adrenal gland influences reproduction in sheep (30–33), however, few studies have assessed the function of lncRNAs in this organ.

In this study we analyze the key candidate lncRNAs and mRNA in the HPA axis that affects seasonal reproduction of Sunite ewes through transcriptome sequencing of the adrenal gland. This provides a new perspective for the study of sheep seasonal reproduction.

## Materials and Methods

### Ethics Statement and OVX + E_2_ Model Building

Ethics approval (No. IAS2018–3) was granted by the Animal Ethics Committee of the Institute of Animal Sciences of Chinese Academy of Agricultural Sciences (IAS-CAAS) (Beijing, China). Nine non-pregnant adult Sunite ewes (aged 2–3 years old; weight 30–40 kg), which were randomly selected from a farm in Bayan Nur City (40°75′north latitude), Inner Mongolia Autonomous Region, China, were used for the study. The ovaries of these animals were removed by surgery, and an estrogen silicone tube was implanted subcutaneously in the neck of the sheep, as described previously (34–36). The ewes were randomly divided into three groups: SP42 (short photoperiod for 42 days; *n* = 3), LP42 (long photoperiod for 42 days; *n* = 3) and SP-LP42 (short photoperiod for 42 days followed by a long photoperiod for 42 days; *n* = 3). The conditions for the long photoperiod were 16 h of light per day and 8 h without light. The conditions for the short photoperiod were 8 h of light exposure and 16 h without light exposure. All sheep had free access to water and feed in an enclosed climate control chamber with only artificial light sources.

### Tissues Acquisition and Sequencing

Adrenal gland tissue from euthanized ewes was quickly preserved in liquid nitrogen with tweezers. The stored tissues were used for RNA extraction with TRIzol Reagent (Invitrogen, Carlsbad, CA, USA) according to the manufacturer's instruction. The purity of the RNA samples was detected by a Nano Photometer^®^ spectrophotometer (IMPLEN, Westlake Village, CA, USA). A Qubit^®^ 3.0 RNA Assay kits (Life Technologies, CA, USA) and RNA Nano 6000 Assay (Agilent Technologies, CA, USA) were used to determine the integrity and concentration of RNA samples. The RNA integrity number (RIN) value of all samples being greater than seven.

The lncRNA library was constructed with 3 μg of high-quality RNA using the NEB Next Ultra Directional RNA Library Prep Kit (NEB, Ispawich, USA) for Illumina, according to the manufacturer's instructions. During this process, Ribo-Zero™ GoldKits (TEANGEN, Beijing, China) were used to remove rRNA. In addition, we used the UNG enzyme to degrade the second strand of U-containing cDNA and performed PCR amplification to obtain the RNA library, RNA-sequencing libraries were generated by paired-end (PE150) sequencing. The RNA library was then sequenced at a concentration of 1 ng/μL RNA using Hiseq 2500 (Illumina, San Diego, CA, United States). All sequencing data was outsourced to Annoroad Gene Technology Co., Ltd. (Beijing, China).

### Data Quality Control and Transcriptome Assembly

Bcl2fastq (v2.17.1.14) is used to process the offline data and convert the original image file into raw sequencing reads on base calling, that was raw read. Clean reads were acquired using in-house Perl script made by Annoroad Genentech Co., Ltd. (Beijing, China) from the raw reads through the removal of: reads with adaptor contamination (i.e., adaptor reads with more than five contaminated bases), low-quality reads (i.e., more than 50% of the bases in the reading have a mass Phred Quality Score of *q* ≤ 19), reads with a rate of N > 5% (i.e., for double-end sequencing, if one-end sequencing does not meet the above requirements, the reads of both ends are removed), and those that matched with ribosomal RNA. We used the *Ovis aries* reference genome (Oar_v4.0), and the genome annotation file from ENSEMBL. Clean reads were then mapped to the reference genome using HiSAT2 (v2.0.5) (37) and StringTie (v1.3.2d) was used to assemble the transcripts (38). HiSAT2 was run with “-rna—strandness RF” and “-dta -t -p 4,” String Tie with “-G ref.gtf -rf−1,” and the other parameters were set as the default.

### lncRNAs and mRNAs Identification and Differential Expression Analysis

Novel lncRNAs transcripts were identified on the following conditions: its length is ≥200 bp, the number of exons is ≥2, and its reads coverage is >5. And remove the known mRNA and other non-coding RNA of the species. Importantly, the coding-non-coding index (CNCI) (39), the coding potential calculator (CPC) (40), the protein families database (PFAM) (41), and the coding potential assessment tool (CPAT) (42) software was used to determine if the transcripts had coding potential and whether they were new transcripts. CNCI was run with “–score 0 –length 199—exon_num 2” with the other parameters set as the default. In both CNCI and CPC, a score <0 was considered to indicate that the lncRNA could be defined as a non-coding RNA. Pfam was run with “minimum protein length: 60” and the other parameters set as the default. CPAT (v1.2.1) was used to screen the coding RNAs by constructing a logistic regression model and calculating Fickett score and Hexamer score, which were based on open reading frame (ORF) length and coverage, respectively.

We used the HTSeq Python package (v0.6.1) to calculate read counts, HTSeq was run with “-I gene_id -f bam -s” and “reverse -a 10 -q” with the other parameters set as the default. DESeq (43) was then applied to identify the differential expression of the lncRNAs based on the normalized counts by using three comparisons: SP42 vs. LP42?SP42 vs. SP-LP42 and LP42 vs. SP-LP42. In addition, |Log2Ratio| ≥ 1 and *q* < 0.05 was considered to be screening threshold of significantly differential expression. The fragments per kilobase per million mapped reads (FPKM) were calculated to represent the expression levels of the lncRNAs and mRNAs (44). Based on the log2 (FPKM) value of mRNA and lncRNA, clustering analysis was performed using pheatmap (v1.0.2) to explore the similarities and analyze the relationships between the different libraries (45). The analysis consisted of Pearson's correlation and Euclidean distance methods.

### Target Gene Prediction of lncRNAs and Gene Enrichment Analysis

To better understand the function of differentially expressed lncRNAs in SP42 vs. LP42, SP42 vs. SP-LP42 and LP42 vs. SP-LP42 we carried out target gene predictions. The target genes can be divided into cis-targets and trans-targets based on the distances and expressions correlation of lncRNAs and protein-coding genes. When the expression quantity correlation coefficient of a lncRNA, and its corresponding target mRNA, was ≥ 0.95 it was considered to be a potential trans-target. If the lncRNAs were located < 50 kb from nearby genes we assigned cis-targets function to them (24).

We performed Gene Ontology (GO) and Kyoto Encyclopedia of Genes and Genomes (KEGG) analyses by using the clusterProfiler package (v3.16.0) to clarify the potential roles of the targeted genes of differentially expressed lncRNAs. The hypergeometric test method was applied to assess significantly enriched GO terms and KEGG pathways. Those with false discovery rate (FDR) < 0.1 and *q* < 0.05, were considered to be significantly enriched.

### Construction of Integral lncRNA–mRNA Interaction Networks

The regulatory network analysis of differentially expressed lncRNAs, and target genes, was drawn according to the relationship between the differentially expressed lncRNAs and mRNA genes, and the genes predicted by cis- and trans-targets of lncRNAs using Cytoscape software.

### Data Validation

Transcripts (*n* = 8) were randomly selected and the primers were designed by primer 5.0 software. The designed primers were synthesized by Beijing Tianyi Huiyuan Biological Technology Co., Ltd. The qPCR reaction conditions were as follows: 95°C for 15 min, followed by 40 cycles of 95°C for 10 s and 60°C for 30 s. The data obtained from the qPCR reaction was evaluated using the 2^−ΔΔCt^ method and statistically analyzed using a one-way analysis of variance in the SPSS20.0. The results are presented as means ± standard deviation. *p* < 0.05 was considered statistically significant.

## Results

### Identification of lncRNAs and mRNAs in the Adrenal Gland Tissue

**Figure 1:**
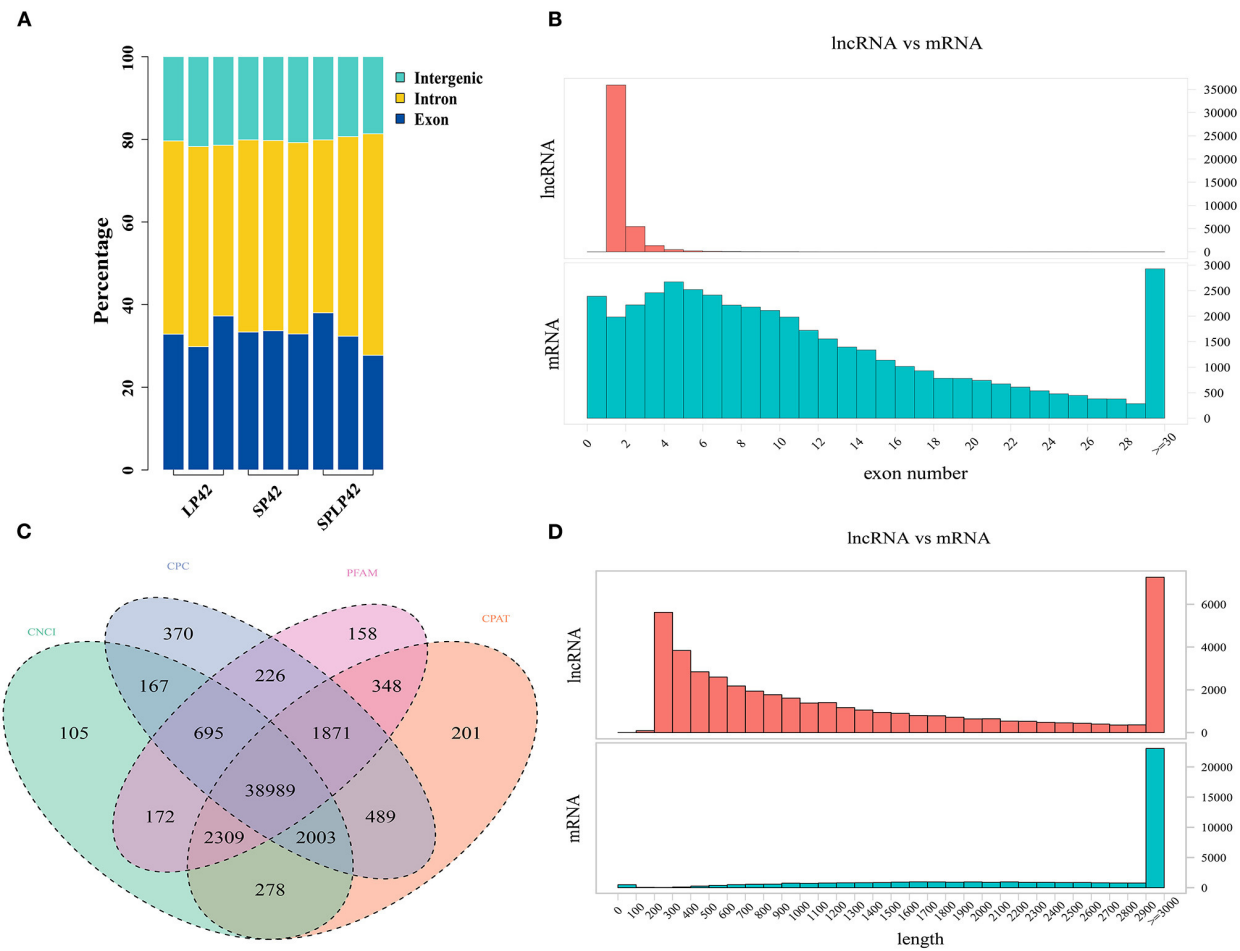
Identification of lncRNAs and mRNA in sunite sheep adrenal gland. **(A)** The regions of identified long non-coding RNAs (lncRNAs) in short photoperiod for 42 days (SP42), long photoperiod for 42 days (LP42), and short photoperiod for 42 days and turn to long photoperiod for 42 days (SP-LP42). **(B)** The exon number of lncRNA and mRNA. **(C)** The results of novel lncRNA predictions by using CNCI, CPC, PFAM, and CPAT software tools. **(D)** The length of lncRNA and mRNA.

The RNA-Seq raw data obtained in this study were subjected to quality control. The results are shown in Table 1 and Supplementary Table 1. In total, SP42 (*n* = 117,038,475), LP42 (*n* = 108,176,645) and SP-LP42 (*n* = 117,740,833) clean reads of average were obtained, respectively, from adrenal gland tissues. Q30 base rate as the filtered data standards, the results show that the percentage of each sample more than 93.70%, above suggests that higher credibility. In comparison with the reference genome (Oar_v4.0) of *Ovis aries*, the mapping rate of each sample is >94%, which is a satisfactory sequencing results. Subsequently, regions in the genome with the identified lncRNAs were predicted (Figure 1A). We found that many of the lncRNAs belong to intron regions, followed by exon and intergenic regions (Supplementary Table 1). In addition, many of the lncRNAs were longer than 200 bp, with many in the range of about 2,900–3,000 bp in length, and the majority of lncRNAs have only two exons. Compared with lncRNAs, mRNAs have more than two exons on average, and most of the lengths are concentrated in the range of 2,900–3,000 bp (Figures 1B,D). We also identified novel lncRNAs by using CNCI, CPC, PFAM and CPAT software to predict the screened non-coding RNA. The results reveal that 38,989 novel lncRNAs were identified and that 29,695 novel lncRNAs were expressed in our samples, including lncRNAs (*n* = 10,362), antisense lncRNAs (*n* = 2,462) and intronic lncRNAs (*n* = 16,871) (Figure 1C; Supplementary Table 2).

**Table 1:** Summary of the mapping data from the adrenal gland tissues.

**Items**	**Raw reads number**	**Clean reads number**	**Mapping rate**	**Clean Q30 bases rate (%)**	**Multimap rate**
SP42A1	129,756,938	125,490,436	95.46%	94.07%	8.73%
SP42A2	113,449,408	110,469,132	94.98%	94.07%	6.43%
SP42A3	118,849,666	115,155,856	95.25%	94.03%	8.07%
LP42A1	115,231,936	111,648,952	94.94%	94.22%	7.50%
LP42A2	106,757,808	101,268,666	94.35%	94.53%	6.13%
LP42A3	115,480,206	111,612,316	95.27%	94.03%	8.73%
SPLP42A1	124,707,824	121,476,492	94.71%	94.19%	6.65%
SPLP42A2	123,408,246	120,023,448	95.13%	94.14%	7.05%
SPLP42A3	117,474,594	111,722,560	94.96%	93.70%	6.66%

*LP42, and SP42 represent a long light period every day for 42 days and a short light period every day for 42 days. SP-LP42 means short light period every day for 42 days, followed by a long light period for 42 days. A represents the adrenal gland in the sample ID*.

### Differential Expression Analysis of lncRNAs and mRNAs

**Figure 2:**
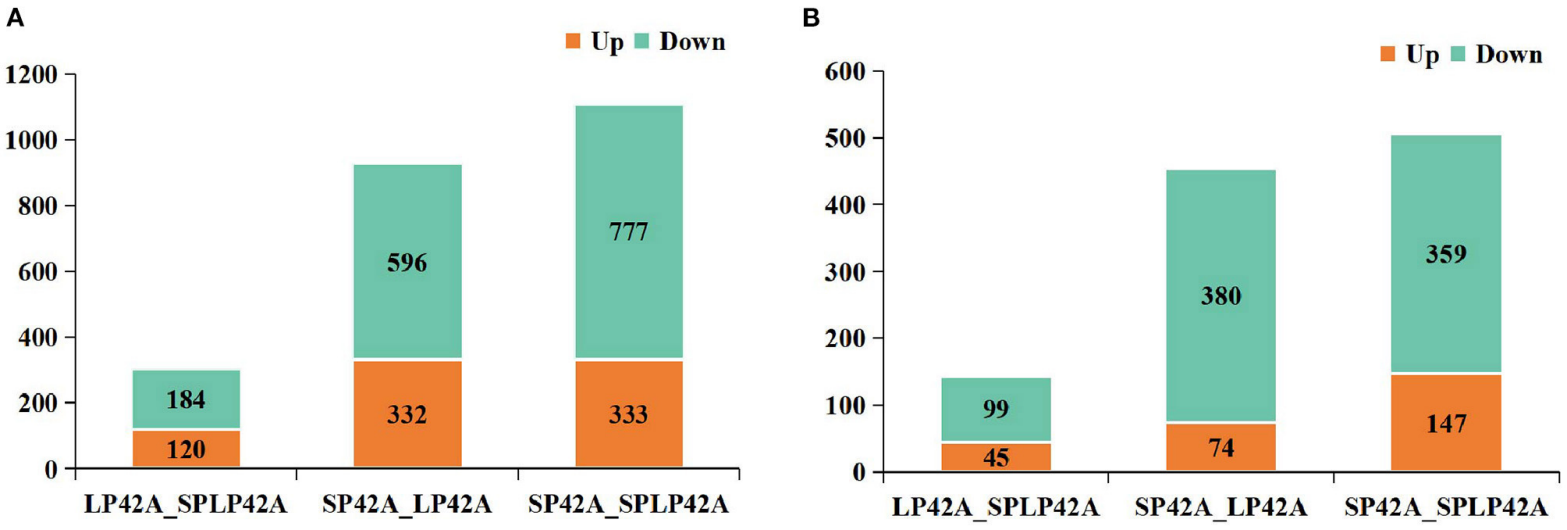
The histogram of DELs and DEMs in different comparable groups. **(A)** DELs **(B)** DEMs. Where red and green represent up- or down regulation, respectively.

**Figure 3:**
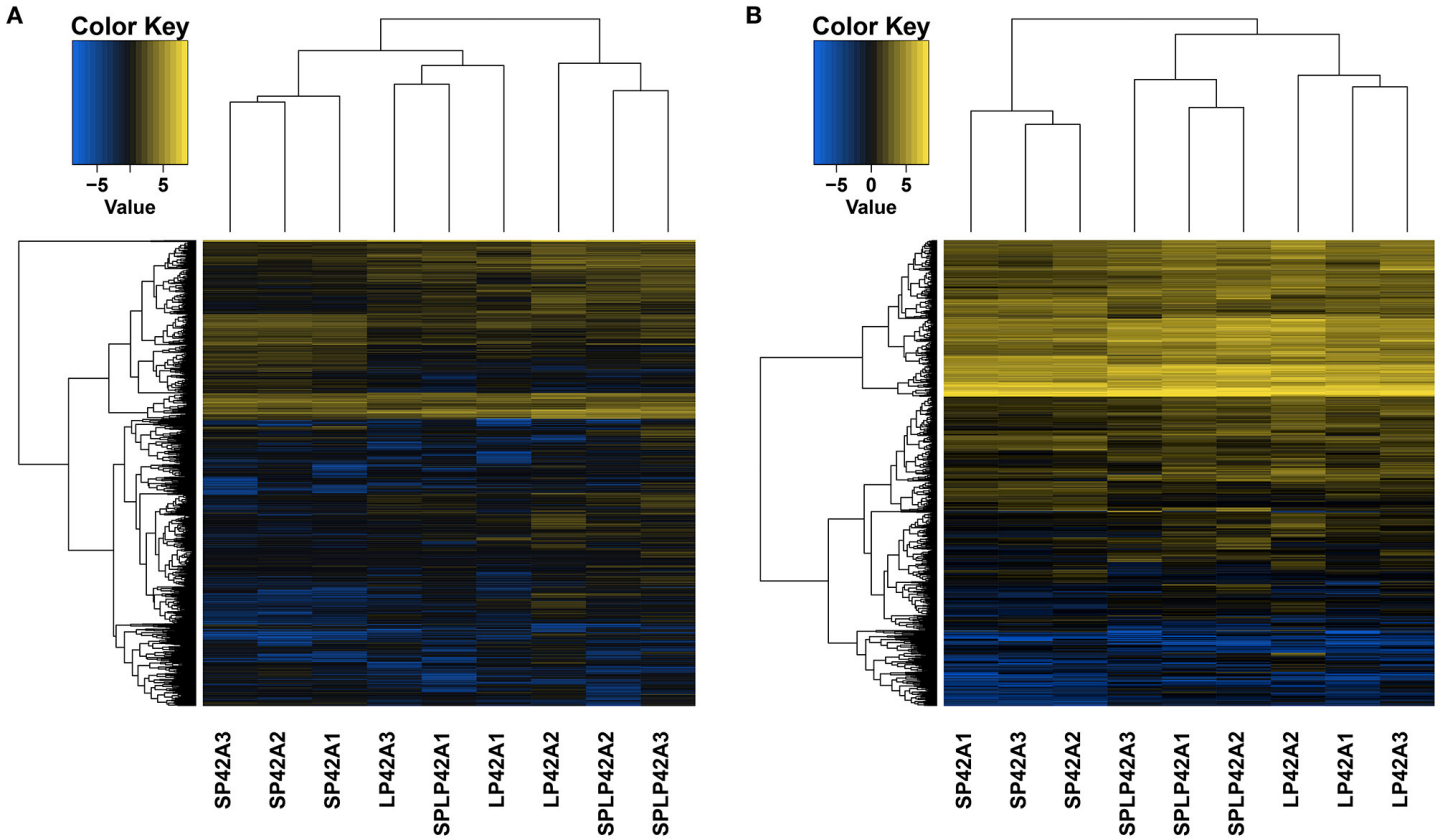
The differentially expressed lncRNAs and mRNAs in each sample using Heat maps. According to a base logarithm of 2 of expression about DEMs and DELs in each sample and the Euclidean distance was calculated, and then the overall clustering results of the samples were obtained by systematic clustering method (Hierarchical Cluster), **(A)** lncRNAs, **(B)** mRNAs.

Pursuant to the expression of differentially expressed lncRNA and mRNA (DELs, DEMs) in each sample, |log2Ratio| ≥ 1 and *q* < 0.05 as cut-off, we found 304, 1,110 and 928 DELs in LP42 vs. SP-LP42, SP42 vs. SP-LP42 and SP42 vs. LP42, respectively. The number of up-regulated genes was 120, 333 and 332, respectively, and the number of down-regulated genes was 184, 777 and 596 respectively. We also identified 144 DEMs (up-regulated 45, down-regulated 99) in LP42 vs. SP-LP42, 454 DEMs (up-regulated 74, down-regulated 380) in SP42 vs. LP42, and 506 DEMs (up-regulated 147, down-regulated 359) in SP42 vs. SP-LP42 (Figure 2; Supplementary Table 3). According to a base logarithm of 2 of expression about DEMs and DELs in each sample and the Euclidean distance was calculated, and then the overall clustering results of the samples were obtained by systematic clustering method (Hierarchical Cluster; Figure 3). An interesting phenomenon about the pattern of DELs is that cluster analysis showed SP-LP42A1 and LP42A2 as mixed groups, and the pattern of DEMs showed perfect groups which is divided into three parts (Figures 3A,B). As we expected, there were significant differences in DELs and DEMs between SP42 treated group and LP42 treated group. However, the expression pattern of DELs indicates that there may be a similar pattern between SP-LP42 and LP42, but the reason is not clear. This, therefore, led to subsequent mining key candidate lncRNA and mRNA transcripts mainly concentrated in the SP42 vs. LP42 comparison group.

### Validation of RNA Sequencing Using RT-qPCR

**Figure 4:**
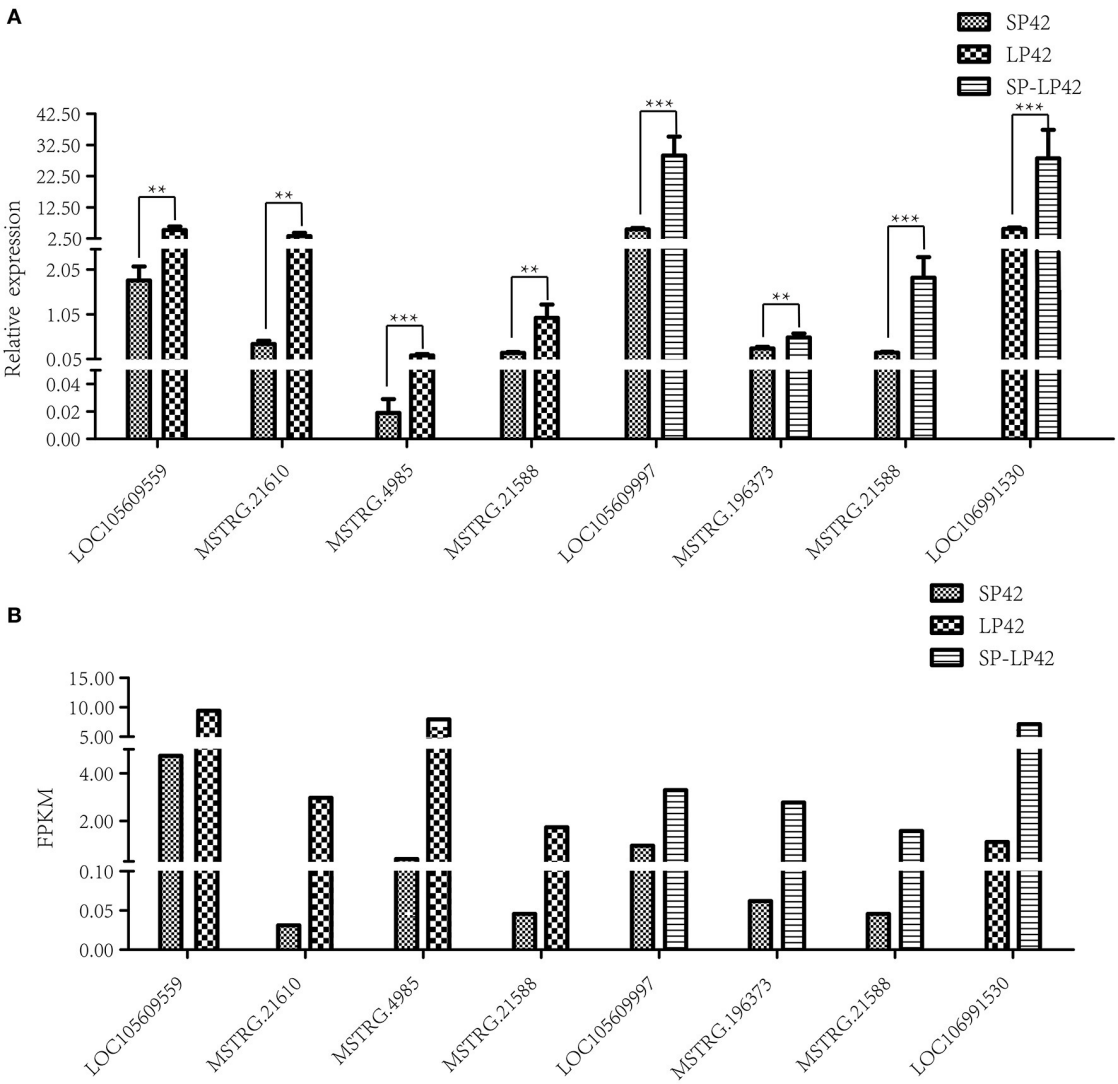
Validation of RNA-Sequencing (RNA-Seq) data using reverse transcription real-time quantitative polymerase chain reaction (RT-qPCR). Different types of rectangles represent different light period processing. The figure ** and *** represents the *p* value ≤ 0.05 and 0.01, respectively. **(A)** RT-qPCR, **(B)** RNA-seq.

To verify the sequencing reliability, seven lncRNAs (Table 2) were randomly selected from the three comparison groups and subjected to RT-qPCR testing. The relative gene expression was calculated using the 2^–ΔΔCt^ method. The results found similar expression patterns using RNA-Seq and RT-qPCR (Figure 4).

**Table 2:** Real-time quantitative polymerase chain reaction primers and sizes of the amplification products of the selected lncRNAs and housekeeping genes.

**lncRNA**	**Primer sequence (5^**′**^-3^**′**^)**	**Product size (bp)**
LOC106991530/ XR_003591261.1	CTCCGGGAAACTTGGTCTCT	89
	CCCAGTTCTGCCAGGAGTTA	
LOC105609559	GAAGCCACCCAAATCCAGAC	182
	GAGCACCAACCATCCTCTCT	
LOC105609997	TGGCTTCCATGGACTGATGT	174
	AATCCACACTCCTCCCTTGG	
MSTRG.21610	GCGGAGGAAGTAGGCTCTAG	167
	CTCGCATCCAAAGCTCAGAC	
MSTRG.4985	AGGAAATCAACAACGGTGCC	80
	TTCCACGTTTCCTCTCCCTC	
MSTRG.21588	GCTATAGGAAGGGCTCTGGG	97
	GGCACAACTGAAGCAACTGA	
MSTRG.196373	CCGTGAACTTGGTGGCATAG	191
	TTCTCCTACCTGCCTCCTCT	
β-actin	CCAACCGTGAGAAGATGACC	97
	CCCGAGGCGT ACAGGGACAG	

### Gene Enrichment Analysis

**Figure 5:**
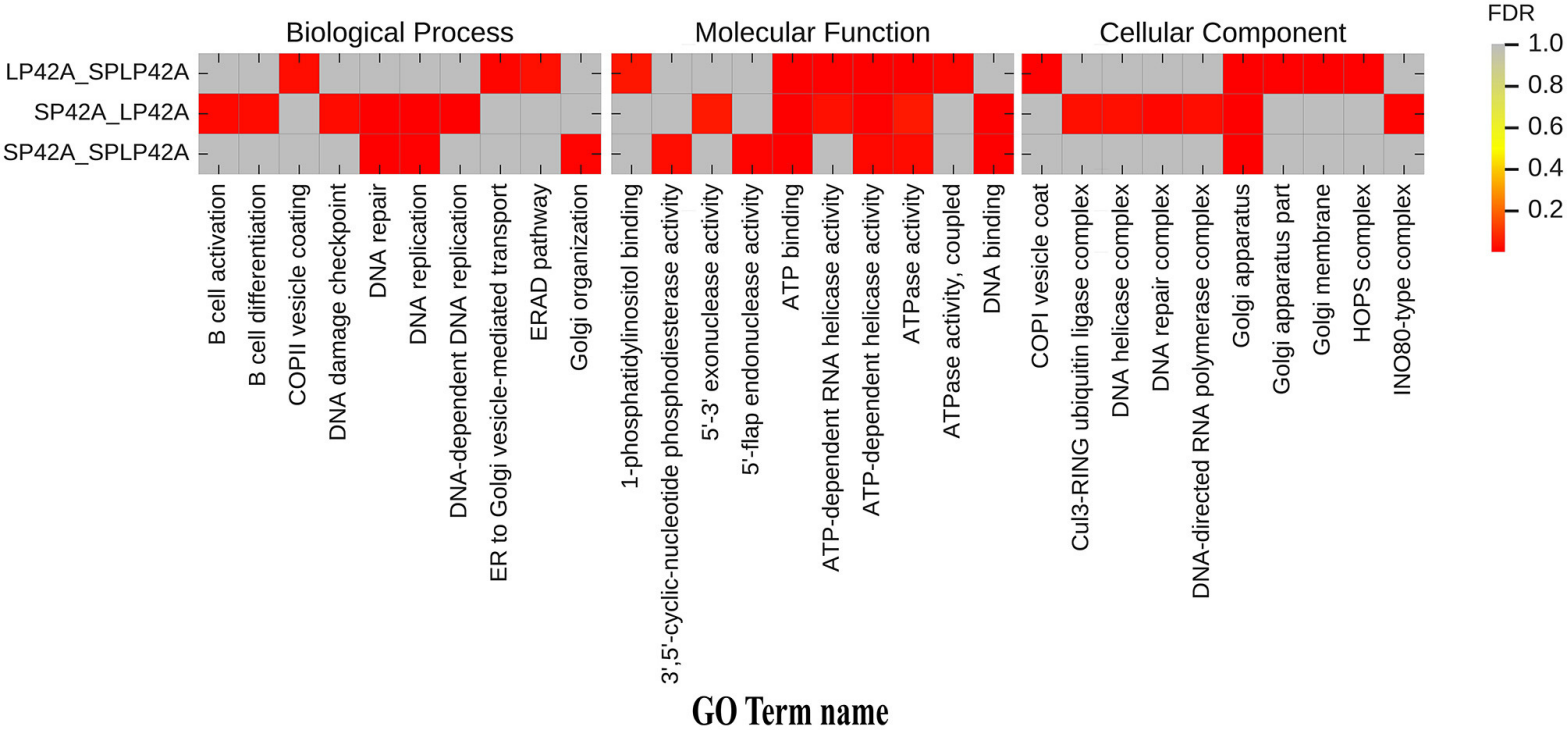
Histogram of GO enrichment of target gene of DELs. Heat maps showing the GO items enriched in the three comparison groups of LP42 vs. SPLP42, SP42 vs. LP42 and SP42 vs. SPLP42.

**Figure 6:**
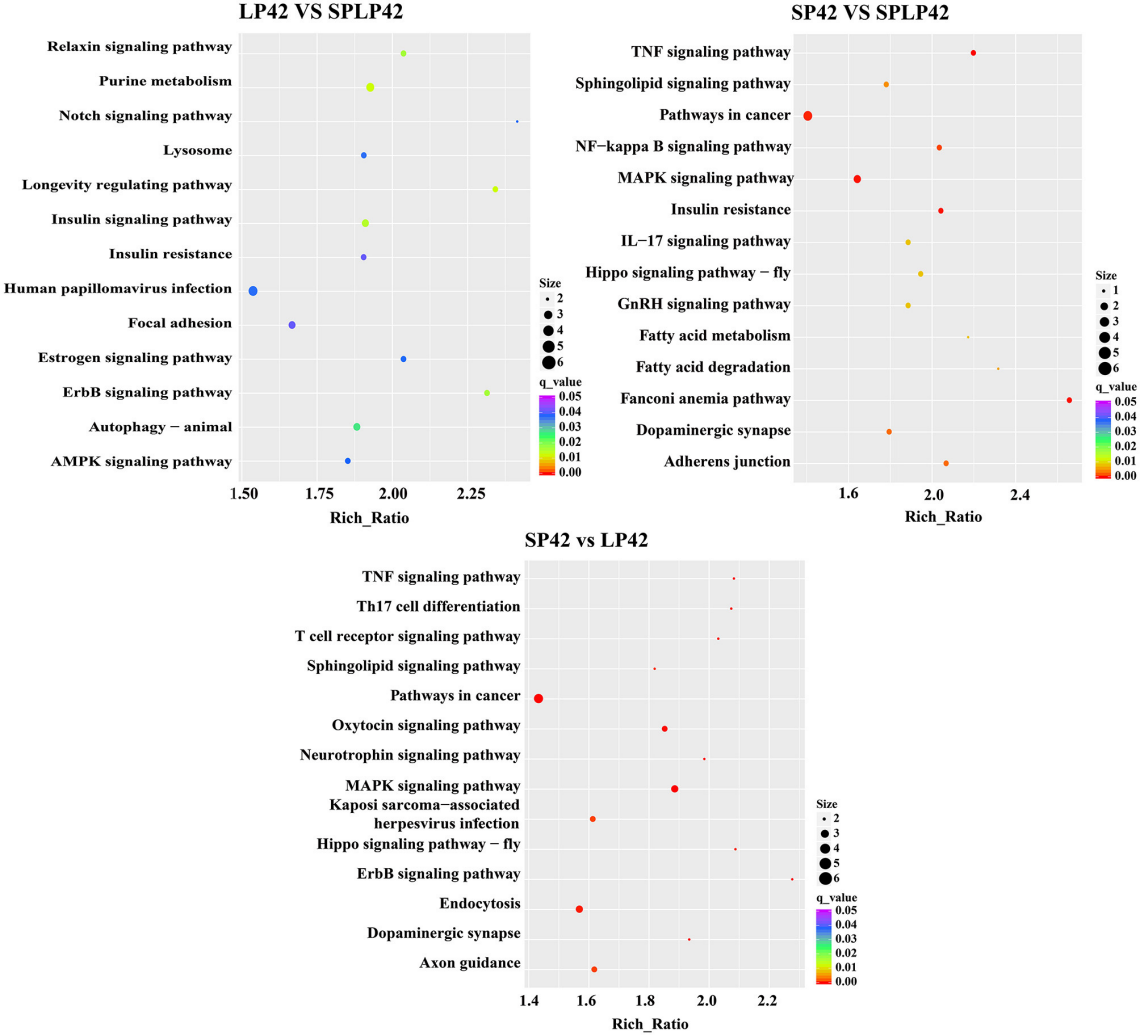
KEGG pathway enrichment analysis of target gene of DELs. The significant enriched KEGG pathways in the three comparison groups of LP42 vs. SPLP42, SP42 vs. LP42 and SP42 vs. SPLP42.

GO annotation and KEGG enrichment analysis were conducted using the identified target genes of DELs. Many GO terms related to ATP binding, Golgi organization, ATP-dependent helicase activity and ATPase activity (Figure 5; Supplementary Table 4). However, the KEGG pathway enrichment of the LP42 vs. SPLP42 group was not as significant as that of the other two groups. The SP42 vs. SPLP42 group, and the SP42 vs. LP42 group, were shown to have similar pathways. Pathways associated with these two groups were related to TNF signaling, sphingolipid signaling, cancer, MAPK signaling, Hippo signaling and dopaminergic synapse (Figure 6; Supplementary Table 5).

### Building lncRNA–mRNA Interaction Networks

**Figure 7:**
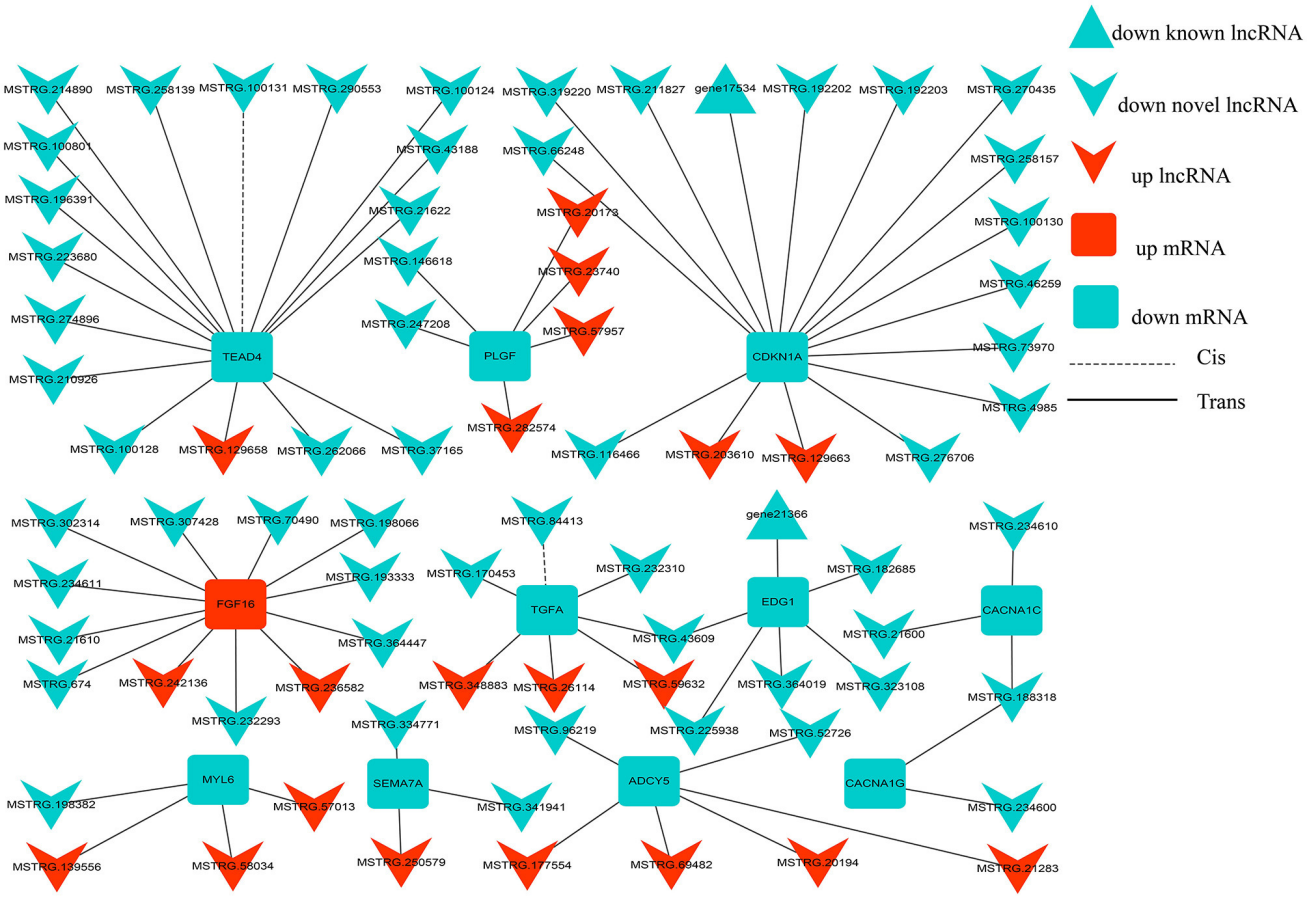
lncRNA-mRNA interaction networks. The interaction networks of lncRNAs and their corresponding target genes in SP42 vs. LP42, where the solid and dashed lines represent trans- and cis-regulation functions, respectively; red and green represent up- and downregulation, respectively; and round rectangle, V and triangle represent mRNAs, novel lncRNAs and known lncRNA, respectively.

To further describe the interaction between lncRNA, and its target genes, we constructed an interaction network of differentially expressed genes in the SP42 vs. LP42 comparison group. A lncRNA/mRNA co-expression network was constructed using 82 differentially expressed lncRNAs and 11 target genes involved in reproductive-related pathways (Figure 7). Twenty up-regulated lncRNA, and 60 down-regulated lncRNA, were identified. Of these, only 2 of the 60 down-regulated RNA are known lncRNAs. The remainder is novel lncRNA. *FGF16* is the only up-regulated gene (of the 11 target genes), and the rest of the target genes are down-regulated. Interestingly, only two lncRNA have a cis-regulatory relationship with their target genes. The remainder is trans-regulatory relationships.

## Discussion

The influence of long non-coding RNA on the reproductive function of sheep had been extensively investigated. Several genes that affect sheep reproduction had been found in lncRNA studies of the hypothalamus (29) and adrenal tissue (33). The hypothalamus was the center that regulates the life activities of mammals, including survival, growth and development, and reproduction. Our team had conducted in-depth research on the hypothalamus and ovaries (46). It was well-known that seasonal estrus is the key factor affecting sheep reproduction, but the change of photoperiod was the key factor affecting the change of seasonal estrus rhythm (9, 10). Photoperiod could be considered as a source of exogenous stress in animals, and the adrenal gland is a key organ to deal with stress response. The OVX + E_2_ model was a good model to study the effect of light period on reproduction (21, 22, 47), Therefore, we used this model for transcriptome sequencing analysis.

We detected a large number of lncRNA and mRNA in the adrenal gland of Sunite sheep by RNA-Seq and counted the length, and the number of exons. We found that the length of lncRNA was less than that of mRNA. Some studies had shown that the length, and exon number, of lncRNA in the sheep hypothalamus was larger than that of the goat hypothalamus (26, 48). Further studies had found that the lncRNA length of the sheep hypothalamus was also longer than that of mice, however, the number of exons was less than that of mice (29, 49). Our study found that the length, and exon number, of lncRNA and mRNA, in sheep adrenal tissue was different from sheep hypothalamus tissue. In particular, the regions of identified lncRNA were significantly different from that of other sheep hypothalamus, more lncRNA were clustered in an intron, followed by exon and intergenic and the type of intronic RNA accounts for more than 56.8% (46). Therefore, lncRNAs were tissue- and species-specific (50).

Cluster analysis of lncRNAs and mRNAs showed that the three samples of the SP42-lncRNA group and all samples of mRNAs were perfectly clustered together, respectively, but the lncRNA of LP42 group and the SP-LP42 group were not completely classified. Thus, did it mean that the similarity of lncRNA expression between the LP42 group and the SP-LP42 group? Is it because the variation and restoration of photoperiod mode also leads to the change and restoration of lncRNA expression pattern? Is SP42 vs. SP-LP42 consistent with the differentially expressed genes of SP42 vs. LP42? To answer these questions, we carried out GO and KEGG enrichment analysis. We found our inference was correct, the SP42 vs. LP42 comparison group and the SP42 vs. SP-LP42 comparison group in the case of *q* ≤ 0.01, GO term and KEGG pathway are the same. However, LP42 vs. SP-LP42 did not find KEGG pathway with *q* ≤ 0.01. Therefore, we selected the pathway with significant enrichment of KEGG in the SP42 vs. LP42 comparison group to screen candidate genes affecting reproduction.

The fibroblast growth factor 16 (*FGF16*) gene was related to oocyte maturation. In dairy cows the expression of the *FGF16* gene was correlated to oocyte quality (51). In summer, when oocyte quality was low, the expression of *FGF16* was low. Conversely, in winter, when oocyte quality was high, the expression of *FGF16* was high. We found that the expression of *FGF16* gene was up-regulated in the comparative group of SP42 vs. LP42; indicating, that in adrenal tissue, the expression of *FGF16* gene in long photoperiod was nearly 10 times lower than that in short photoperiod. Finally, two important up-regulation of *FGF16* gene lncRNA (MSTRG.242136, MSTRG.236582), and 11 down-regulation of lncRNA were identified in our study. Among them, the expression abundance of MSTRG.176476 lncRNA was the highest, and |Log2 Fold Change| was about 2. In addition, although the expression abundance was not high, the largest |Log2 Fold Change| close to 7 is MSTRG.21610 lncRNA, but its effect on *FGF16* gene expression remains to be further verified.

Placental growth factor (*PLGF*) was another gene that may be associated with reproductive function, most notably with embryo implantation (52, 53). *PLGF* was a member of the vascular endothelial growth factor family of proangiogenic factors regulated angiogenesis and microvessel density (MVD) (54, 55). Moreover, the serum level of *PLGF* had been positively correlated with fecundity in Hu sheep (56). The differential expression of *PLGF* gene in the SP42 vs. LP42 comparison group was also found in our study, and |Log2 Fold Change| was close to 2.4. As we know that the retina was the window for receiving light signals and was filled with microvessels. Study had shown that *PLGF* was related to retinal angiogenesis (57). Therefore whether *PLGF* gene affected light signal reception through related pathways, and thus, affected seasonal estrus needs, need to be further explored. Interestingly, the differentially expressed gene cyclin-dependent kinase inhibitor 1A (*CDKN1A*), which was found in our study and |Log2 Fold Change| was close to 1.6, *p* ≤ 0.01, played an important role in the apoptosis of vitreous microvascular epithelial cells (58). At the same time, another key candidate gene Endothelial differentiation gene1 (*EDG1*) for angiogenesis was also found in our study and |Log2 Fold Change| was close to 1.4, *p* ≤ 0.01. Specifically, *EDG1* also known as sphingosine 1–phosphate receptor 1 (*S1PR1*) which belong to the rhodopsin family, was involved in angiogenesis. This family had been considered to be typical members of the rhodopsin superfamily. The function of most opsins was split into two steps: light absorption and G-protein activation. In addition, *EDG1* expression had been observed in ovarian tissues and the family of *S1PR1* also had been reported to play an important role in ovarian angiogenesis, suggesting that the *EDG1* signal may regulate ovarian angiogenesis. Generally, ovarian angiogenesis seems to be one of the factors responsible for follicular development. Consequently, *EDG1* was currently used as a genetic marker for reproductive traits in cattle because there was a significant correlation between *EDG1* polymorphism and the age of first birth in cattle (59).

In our experimental design, photoperiod as a unique variable and the only exogenous stress, the experimental samples only through the retina to receive light stimulation to change the biological clock to further change their hormone secretion. Then whether it is possible to explore the secretion of seasonal estrous hormones according to the mechanism of the retina may be a valuable topic. It was well-known that GnRH played an important role in sheep reproduction. Among the differential genes identified, semaphorin 7 (*SEMA7A*), |Log2 Fold Change| was close to 1.4, was reported to be closely related to the development of mouse GnRH-1 neurons system (60). Among the differential genes identified by us, the gene with the similar function was voltage-dependent calcium channel L type alpha-1C (*CACNA1C*), which had been proved to be a key candidate for precocious puberty in Jining gray goats (61). Interestingly, the gene had also been shown to be closely related to GnRH. It co-ordinatively participated in ERK activation and caused the increase of FSH and LH secretion in the GnRH signal pathway (62). In addition, adenylate cyclase 5 (*ADCY5*) which |Log2 Fold Change| was close to 2 in our study had also been proved to be a key candidate gene affecting the fecundity of dairy cows (63). Thus, whether the gene affects the reproductive ability of sheep through the adrenal gland under different light conditions needs to be further explored.

## Conclusion

In conclusion, this study provided lncRNA and mRNA expression profiling in the adrenal gland of sheep during different photoperiods. Several photoperiod-induced targeting key genes of seasonal reproduction (*FGF16, PLGF, CDKN1A, SEMA7A, EDG1, CACNA1C* and *ADCY5*) were predicted in the adrenal gland of sheep. These results may provide a solid molecular basis for follow-up studies on seasonal estrus in sheep.

## Data Availability Statement

The datasets presented in this study can be found in online repositories. The names of the repository/repositories and accession number(s) can be found in the article/Supplementary Material.

## Ethics Statement

The animal study was reviewed and approved by the Science Research Department (in charge of animal welfare issues) of the Institute of Animal Science, Chinese Academy of Agricultural Sciences (IAS-CAAS) (Beijing, China) and ethical approval was given by the Animal Ethics Committee of the IAS-CAAS (No. IAS2018-3). Written informed consent was obtained from the owners for the participation of their animals in this study.

## Author Contributions

QiuL and MC designed the research. XD wrote the paper and performed the study. RD, XD, and QinL collected the data. XD and XH analyzed data. MC revised the final manuscript. All authors reviewed the manuscript and approved the final version.

## Funding

This work was financially supported by the National Natural Science Foundation of China (32172704), China Agriculture Research System of MOF and MARA (CARS-38), the Agricultural Science and Technology Innovation Program of China (CAAS-ZDRW202106 and ASTIP-IAS13).

## Conflict of Interest

The authors declare that the research was conducted in the absence of any commercial or financial relationships that could be construed as a potential conflict of interest.

## Publisher's Note

All claims expressed in this article are solely those of the authors and do not necessarily represent those of their affiliated organizations, or those of the publisher, the editors and the reviewers. Any product that may be evaluated in this article, or claim that may be made by its manufacturer, is not guaranteed or endorsed by the publisher.

## Supplementary Material

Supplementary Table 1 The basic information of lncRNA sequencing data.

Supplementary Table 2 The FPKM values of both known and novel lncRNAs in SP42, LP42, and SP-LP42.

Supplementary Table 3 The information of lncRNAs and mRNA expressed in SP42 vs. LP42, SP42 vs. SP42-LP42 and LP42 vs. SP42-LP42.

Supplementary Table 4 GO enrichment annotation for target genes of lncRNAs at the MF, BP, and CC level in SP42 vs. LP42, SP42 vs. SP42-LP42 and LP42 vs. SP42-LP42.

Supplementary Table 5 KEGG enrichment pathway for target genes of lncRNAs in SP42 vs. LP42, SP42 vs. SP42-LP42 and LP42 vs. SP42-LP42.

## Abbreviations

lncRNA: Long non-coding RNA
OVX + E_2_: ovariectomy and estrogen was embedded in the epidermis
HPA: the hypothalamic–pituitary–adrenal
mRNA: message RNA
LP: long photoperiod
SP: short photoperiod
GO: Gene Ontology
KEGG: Kyoto Encyclopedia of Genes and Genomes
LP42: the LP lasts for 42 days
SP-LP42: the SP lasts for 42 days, and then convert it to LP for 42 days
SP42: the SP lasts for 42 days
*FGF16*: fibroblast growth factor 16
*PLGF*: placental growth factor
*EDG1*: Endothelial differentiation gene1
MVD: microvessel density
*S1PR1*: sphingosine 1–phosphate receptor 1
*SEMA7A*: semaphorin 7
*CACNA1C*: voltage-dependent calcium channel L type alpha-1C
*ADCY5*: adenylate cyclase 5.
